# Kurt Jellinger 90: his contribution to neuroimmunology

**DOI:** 10.1007/s00702-021-02358-4

**Published:** 2021-06-10

**Authors:** Assunta Dal-Bianco, Romana Höftberger, Hans Lassmann, Thomas Berger

**Affiliations:** 1 Department of Neurology, Medical University of Vienna, Vienna, Austria; 2 Division of Neuropathology and Neurochemistry, Department of Neurology, Medical University of Vienna, Vienna, Austria; 3 Center for Brain Research, Medical University of Vienna, Vienna, Austria

**Keywords:** Multiple sclerosis, Experimental autoimmune encephalomyelitis, Neuropathology, Neuroimmunology, Alzheimer’s disease, MOG antibody disease

## Abstract

This review honors Kurt Jellinger on his 90th birthday as one of the most outstanding neuropathologists, who has contributed immensely to neuroscience due to his vast experience and collection of excellently documented autopsy cases. Two of his many insightful reports are highlighted here. One report focuses on the pathogenesis of inflammatory demyelinating diseases and investigates the neuropathology in autopsy tissue of a patient, who developed an MS-like disease after repeated treatment with lyophilized bovine brain cells in 1958. More than 60 years later, after reinvestigation of the historic samples in 2015 and subsequent mRNA isolation, next generation sequencing and reconstruction of the antibody, we succeeded in identifying myelin oligodendrocyte glycoprotein (MOG) as the target antigen and provided the missing element between the pathomechanisms in classic EAE animal models and transfer of this disease process into humans. A second significant example of Kurt Jellinger’s contribution to neuroscience was a report on the role of MS in the development of Alzheimer's disease (AD), which found that AD pathology is present to the same extent in demyelinated and non-demyelinated cortical areas in MS and the incidence for AD pathology in elderly MS patients is comparable to the normal-aging population. This indicates that chronic inflammation in the MS cortex alone does not significantly predispose to the development of cortical AD pathology. These and other findings were only possible due to the broad collection of extremely well-defined material established by Kurt Jellinger, which ultimately continues to contribute to translational neuroscience, even decades later.

## Introduction

Kurt Jellinger’s focus expertise is devoted to the clinical and translational neuropathology of neurodegenerative diseases in the central nervous system. However, due to his very broad training and his eminent knowledge and experience covering the entire spectrum of brain diseases, he was an ideal cooperation partner also for research related to inflammatory diseases of the brain and spinal cord. His contributions are manyfold and related to various topics, just to name a few of them on human autoimmune encephalitis (Jellinger et al. 1958; Hochschorner et al. 1990; Bien et al. 2012), on multiple sclerosis (Guseo and Jellinger 1975; Aboul-Enein et al. 2003; Höftberger et al. 2004), on infectious diseases (Seitelberger and Jellinger 1966; Gerstenbrand et al. 1980; Drlicek et al. 1993) and on inflammatory mechanisms in neurodegenerative diseases (Dal Bianco et al. 2008; Jellinger and Wenning 2016). In this short review, we decided to select two examples, illustrating his fundamental work, which provided not only seminal new insights but also sustainably influenced brain research until today: the relation between multiple sclerosis (MS) and autoimmunity and the question whether a chronic inflammatory disease of the nervous system affects neurodegeneration in Alzheimer’s disease (AD).

### Does autoimmune encephalomyelitis in humans lead to multiple sclerosis?

Since the first detailed clinico-pathological descriptions of MS by Rindfleisch (1863), Charcot (1880), Babinski (1885) and Marburg (1906) speculations about the cause of the disease ensued and this even is not settled today. The discussion centers on the “inside-out” versus the “outside-in” hypotheses (Titus et al 2020). The “inside-out” hypothesis postulates that a process within the central nervous system starts a cascade of damage, which triggers a secondary and possibly amplifying inflammatory response. Whether the problem within the brain may be due to a primary neurodegeneration or a (virus) infection is not known. In contrast, the “outside-in” hypothesis postulates that an autoimmune response against targets in the central nervous system is triggered in the peripheral immune system, which then gives rise to inflammation and tissue damage in the central nervous system. The best argument for the “outside-in” hypothesis has been provided by Kurt Jellinger in his report of a unique case (Jellinger et al. 1958). It was already known since the end of the nineteenth century that immunization of humans with a rabies vaccine, which contains brain tissue, can induce an inflammatory disease of the central nervous system (Kortischoner and Schweinburg 1927). Subsequent experimental studies in monkeys induced a similar disease by sensitization with brain tissue, thus suggesting an autoimmune nature of the disease (Rivers et al 1933). In a review of all human cases published before, Stuart and Krikorian (1928) determined that such complication appeared in about one out of 1.000 rabies vaccinated individuals, and that these patients developed either an inflammatory polyradiculoneuritis or an acute disseminated encephalomyelitis. Only, in a very small number of people such immunizations were associated with a pathological phenotype similar to that of MS (Uchimura and Shiraki 1957). Whether this MS- like phenotype was due to autoimmunity alone or in combination with the virus infection or whether MS was just precipitated in a patient susceptible for the disease is still unclear and heavily disputed.

**Fig. 1:**
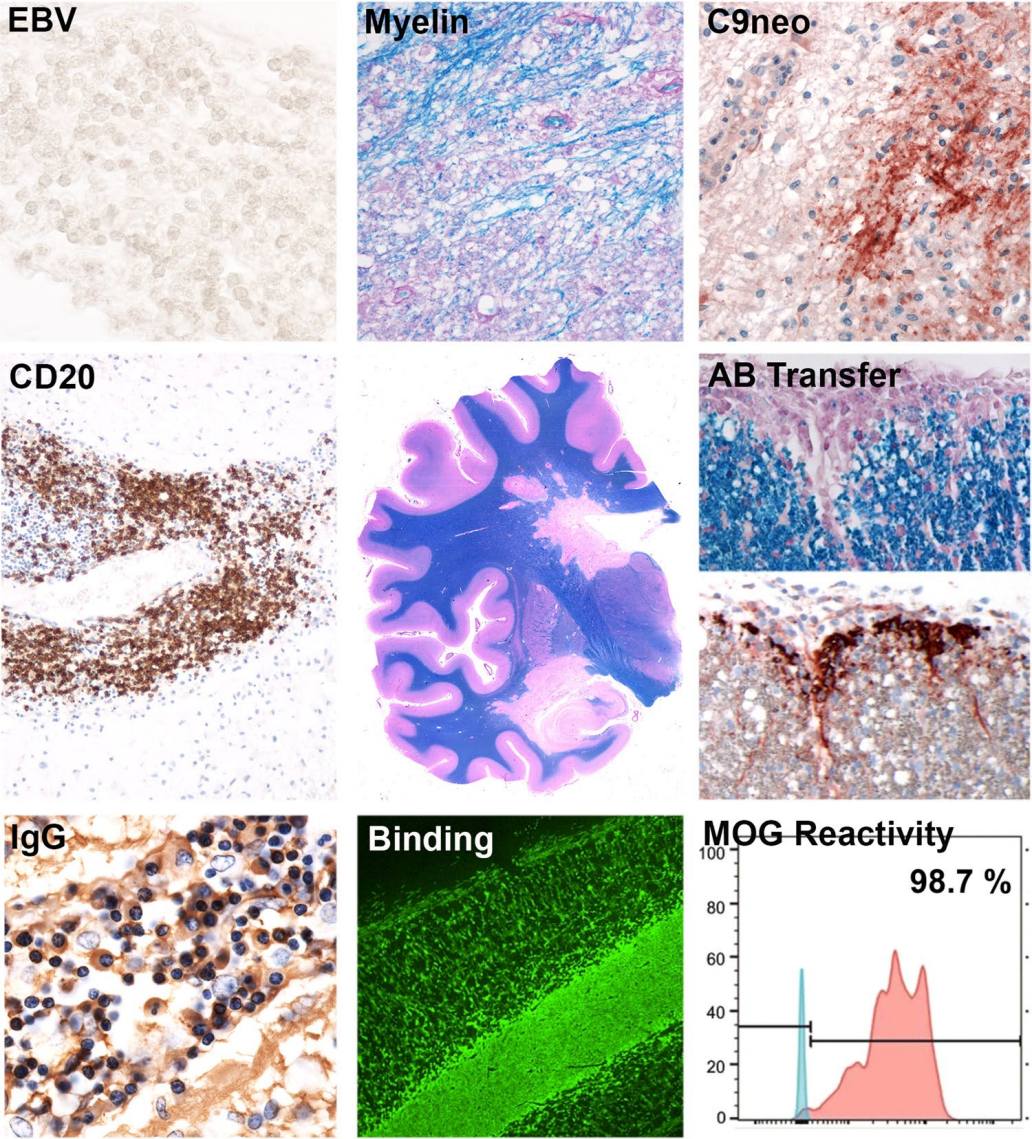
Neuropathology of human autoimmune encephalomyelitis. Top row (left–right): inflammatory cells of the patient are negative for Epstein-Barr virus (EBV in situ hybridization); demyelinated plaques show active demyelination at the lesion edge (Luxol-Fast Blue) and prominent deposition of activated complement (C9neo antigen). Middle row (left–right): Inflammatory cuffs are characterized by abundant CD20^+^ B-cells (CD20); a hemispheric section shows prominent periventricular demyelination with finger-like, perivenous extensions and demyelinated plaques in the cortex and deep gray matter (Luxol Fast Blue); spinal cord sections of Lewis rats with MBP-specific T-cell-induced experimental autoimmune encephalomyelitis and intrathecally injected recombinant antibody of the human autoimmune encephalomyelitis patient show demyelination and deposition of activated complement (upper picture: Luxol Fast Blue; lower picture: C9neo antigen). Bottom row (left–right): plasma cells within the inflammatory cuffs of the patient are positive for IgG (IgG); the recombinant antibody of the patient strongly labels the myelin in commercial slices of primate cerebellum (Euroimmun, Lübeck; Germany; fluorescence labeling with biotinylated anti-human IgG1 and Streptavidin Alexa Fluor 488; green) and recognizes human MOG transfected in COS-7 cells (analyzed by flow cytometry)

The clarification of part of the issue came with the publication of Kurt Jellinger (Jellinger et al. 1958), who first described a similar disease in a patient who died after a misguided therapy using so-called sicca cells. At this time, it was quite popular to follow the approach of “fresh cell therapy” of Paul Niehans to treat patients with fresh animal derived cells from the respective organ, which was affected by the disease, when no other therapeutic option was available (Bohl et al. 1989). In the particular case, described by Kurt Jellinger, the patient received multiple injections of brain and placenta cells for the treatment of Parkinson’s disease. 22 days after the last injection, the patient developed an ascending paresis and died 7 weeks after disease onset in a comatose stage. The pathology revealed an inflammatory demyelinating disease of the central nervous system, which was similar to that seen in patients with Marburg’s type of acute MS. This was a seminal observation which received wide international attention, since it for the first time provided evidence that in humans’ true autoimmunity, induced by active sensitization with neural tissue, may cause a disease of the central nervous system, which mimics MS. However, the search for an MS-specific autoimmune response, performed during the following decades failed (Hohlfeld et al. 2016a, b). For this reason, we recently reinvestigated this case, which fortunately was saved in the archival collection of the former Institute of Neurology, now Division of Neuropathology and Neurochemistry, Dept. of Neurology (Höftberger et al. 2015). Using modern neuropathological techniques, which are currently available for the characterization and typing of MS lesions, we found that the nature of the lesions, the primary demyelination and the neuronal, axonal and glial pathology was indistinguishable from that seen in other cases of acute MS. The only difference was that the inflammatory infiltrates were massively biased toward CD20 positive B-cells. We therefore decided to further characterize the B-cell response in this case. We were lucky to extract sufficient amounts of mRNA from the formalin fixed and paraffin embedded autopsy tissue to resurrect the pathogenic autoantibody, produced by clonally expanded plasma cells in the inflammatory demyelinating lesions by deep sequencing and bioinformatic alignment of the RNA fragments (Beltran et al. 2020). The antibodies recognized a conformational epitope of myelin oligodendrocyte glycoprotein (MOG) and the reconstructed recombinant autoantibody-induced demyelination in rodents after passive transfer (Fig. 1).

This result was highly surprising, since it provided formal evidence in this particular case for an autoimmune encephalomyelitis and the final diagnosis was not MS but MOG antibody associated inflammatory demyelinating disease (MOGAD), which differs from MS by its clinical presentation and its response to therapy (Reindl et al. 2013; Jurynczyk et al. 2017). Still, this case raises important questions, since its pathology is much closer to that of MS than that of MOGAD (Höftberger et al. 2020). As soon as technically possible, a similar resurrection of a potential T-cell mediated autoimmune response should also be done. This could provide the final clue on shared or diverse immune mechanisms between MOGAD and MS. Whatever will be the outcome of these future studies, the research efforts described above are an impressive example, how much one can learn from a single case, when its unusual features are properly recognized by an experienced neuropathologist.

### What does multiple sclerosis tell us about Alzheimer’s disease?

**Fig. 2:**
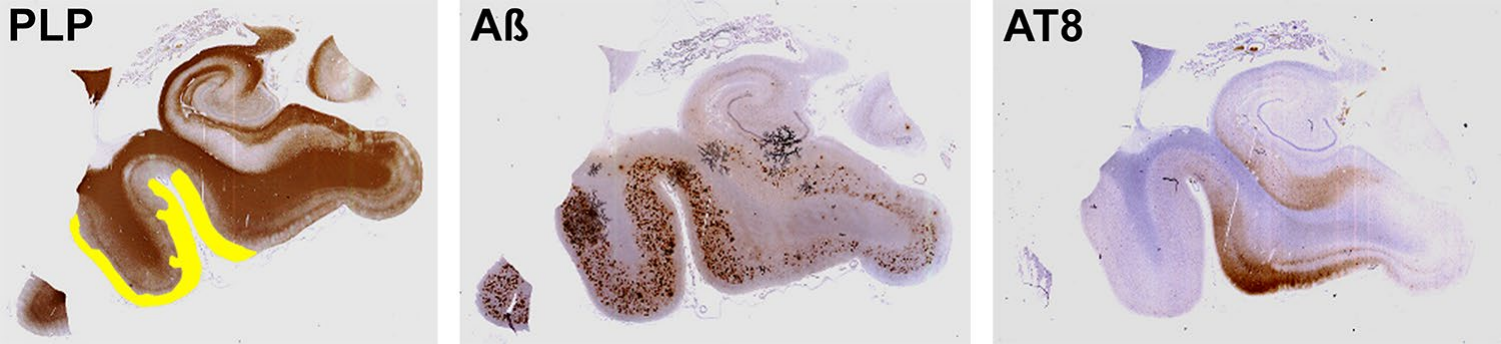
Alzheimer’s disease pathology and multiple sclerosis. Left–right: demyelinated regions (marked in yellow; PLP) in the temporal cortex of an MS patient showing AD pathology with ß-amyloid plaques (Aß) and neurofibrillary tangles (AT8). AT8 = Phospho-Tau-Antibody

Following the traditional view of neuropathology on MS and AD one could question the rationale to look into a potential interaction between these diseases. MS is a disease of young adults and it is a well-defined immune mediated inflammatory disease which damages primarily myelin and oligodendrocytes, while AD affects the aging population and is a neurodegenerative disease of the cerebral cortex. Nevertheless, the diseases share important features. Neurodegeneration is a prominent aspect of MS pathology, in particular in patients with progressive disease (Titus et al 2020). On the other hand, several data suggest a prominent role of neuroinflammation in the pathogenic cascade of AD (Aisen et al. 1994; Eikelenboom et al. 1994) and this is also supported by genome wide association studies (GWAS; Griciuc et al. 2021; Carpanini et al. 2021). In addition, in both diseases activated microglia are associated with tissue injury and neurodegeneration (Fig. 2). Finally, the cortical regions, which are most severely affected in AD, such as the entorhinal cortex, the hippocampus and the temporal neocortex are also predilection sites of cortical lesions in MS (Geurts et al. 2007; Rocca et al 2018). Thus, during aging in MS patients AD pathology, when present, develops in cortical areas, which have been affected by chronic inflammation for decades. On this basis, a number of questions can be addressed:

1. Are the patterns of microglia activation similar or different in a chronic adaptive immunity-driven inflammatory condition such as MS from those in a neurodegenerative disease such as AD?
2. Does chronic inflammation and demyelination predispose to AD pathology in cortical areas, which are preferentially affected in both diseases?
3. Does chronic inflammation and microglia activation in the cortex of MS patients protect against AD pathology?

To address these questions is challenging, since it requires access to a reasonable sample of human autopsy cases of MS patients, who died at an age comparable to AD patients and in whom detailed neuropathological data on MS- and AD-related tissue alterations were available. Due to his life-long and uniquely systematic neuropathological work, Kurt Jellinger was able to provide the basis for such a study (Dal Bianco et al. 2008). The results were remarkable and unexpected. It first turned out that the microglia activation profiles were very similar between MS and AD, even when a large panel of markers for different pro- and anti-inflammatory activation stages were applied. This was particularly the case, when microglia from actively demyelinating MS lesions were compared with plaque associated microglia in AD. The result fits well with more recent data, which show a comparable lesion stage-dependent microglia activation in MS, ischemic stroke, neurotrauma and sepsis (Zrzavy et al. 2017, 2018, 2019, 2021). Although these data cannot exclude that with more sophisticated methods of single cell RNA sequencing subtle differences in microglia activation will appear, they suggest that overall, the patterns of microglia activation rather reflect the activity stage of the lesions than the pathogenetic nature of the lesion.

Having established the similarity in microglia activation between these two conditions, we then turned to the question, whether the chronic inflammation in MS influences the appearance and progression of beta-amyloid (Aß) deposition or the accumulation of tau tangles in the cortex (Dal Bianco et al. 2008). In both, the MS and the AD cohort AD pathology in the temporal cortex was only seen in patients over 65 years of age. When we quantified the density of plaques and tangles in the hippocampus and temporal cortex and compared MS patients with age matched non-MS patients, we did not find a significant difference. Finally, we did not find a difference in the number of Aß deposits and neurofibrillary tangles between normal appearing MS cortex and cortical demyelinated lesions. This implied that microglial activation alone, in the absence of a disease-specific trigger, has little or no effect on the development of demyelination or neurodegeneration. These findings raise doubts regarding the significance of inflammatory mechanisms in the pathogenesis of AD. Based on the results of our study, there is little interaction between the chronic inflammatory process, which occurs in MS and the development of Aß and neurofibrillary pathology in AD. An interesting question, which could not be addressed in our study was to what extent the pre-existing MS- and AD-related cortical pathologies synergize in the propagation of cognitive decline or the development of synaptic and dendritic pathology.

## Conclusions

Neuropathology occupies a prominent place in translational neuroscience. Whenever new molecular disease mechanisms, diagnostic tools or therapeutic approaches are discovered, it is the role of neuropathology to validate their significance in the context of the pathological process, which takes place in the nervous system of the patients. However, when this task is not properly performed, misleading results may cause harm to patients or may waste considerable research resources. The key to properly conduct neuropathological assessment is experience. In fact, the more images and cases are stored in the neuropathologists brain, the more accurate will be his account. Kurt Jellinger is one of the outstanding examples of an experienced neuropathologist. Being trained in the tradition of the Institute of Neurology of the University of Vienna, founded by Heinrich Obersteiner and Otto Marburg, he profited from and massively expanded the case collection and made it available for systematic neuropathological studies in the very broad spectrum of neurological and psychiatric disorders. The access to such an extremely well-defined material was and is a prerequisite for all further scientific efforts.

It was, thus, a privilege for us to have Kurt Jellinger as an essential and outstanding teacher and cooperation partner in our research projects.

## Abbreviations

Aß: Beta-amyloid
AD: Alzheimer’s disease
EAE: Experimental autoimmune encephalomyelitis
MOG: Myelin oligodendrocyte glycoprotein
MS: Multiple sclerosis
